# The LEAD (Lung, Heart, Social, Body) Study: Objectives, Methodology, and External Validity of the Population-Based Cohort Study

**DOI:** 10.2188/jea.JE20180039

**Published:** 2019-08-05

**Authors:** Robab Breyer-Kohansal, Sylvia Hartl, Otto Chris Burghuber, Matthias Urban, Andrea Schrott, Alvar Agusti, Torben Sigsgaard, Claus Vogelmeier, Emiel Wouters, Michael Studnicka, Marie-Kathrin Breyer

**Affiliations:** 1 First Department of Respiratory and Critical Care Medicine and Ludwig Boltzmann Institute for COPD and Respiratory Epidemiology, Otto Wagner Hospital, Vienna, Austria; 2 Second Department of Respiratory Medicine, Otto Wagner Hospital, Vienna, Austria; 3 First Department of Respiratory and Critical Care Medicine and Ludwig Boltzmann Institute for COPD and Respiratory Epidemiology, Otto Wagner Hospital, and Sigmund Freud University, Medical School, Vienna, Austria; 4 Respiratory Institute, Hospital Clinic, IDIBAPS, University of Barcelona and National Spanish Network for Respiratory Research (CIBERES), Barcelona, Spain; 5 Institute of Public Health, Environmental and Occupational Medicine, Aarhus University, Aarhus, Denmark; 6 Department of Medicine, Pulmonary and Critical Care Medicine, University Medical Center Giessen and Marburg, Philipps-University Marburg, Marburg, Germany; 7 Department of Respiratory Medicine+, MUMC+, Maastricht University, Maastricht, the Netherlands; 8 Department of Respiratory Medicine, Paracelsus Medical University, Salzburg, Austria

**Keywords:** population based cohort, epidemiology, respiratory diseases

## Abstract

**Background:**

The Lung, hEart, sociAl, boDy (LEAD) Study (ClinicalTrials.gov; NCT01727518; http://clinicaltrials.gov) is a longitudinal, observational, population-based Austrian cohort that aims to investigate the relationship between genetic, environmental, social, developmental and ageing factors influencing respiratory health and comorbidities through life. The general working hypothesis of LEAD is the interaction of these genetic, environmental and socioeconomic factors influences lung development and ageing, the risk of occurrence of several non-communicable diseases (respiratory, cardiovascular, metabolic and neurologic), as well as their phenotypic (ie, clinical) presentation.

**Methods:**

LEAD invited from 2011–2016 a random sample (stratified by age, gender, residential area) of Vienna inhabitants (urban cohort) and all the inhabitants of six villages from Lower Austria (rural cohort). Participants will be followed-up every four years. A number of investigations and measurements were obtained in each of the four domains of the study (Lung, hEart, sociAl, boDy) including data to screen for lung, cardiovascular and metabolic diseases, osteoporosis, and cognitive function. Blood and urine samples are stored in a biobank for future investigations.

**Results:**

A total of 11.423 males (47.6%) and females (52.4%), aged 6–80 years have been included in the cohort. Compared to governmental statistics, the external validity of LEAD with respect to age, gender, citizenship, and smoking status was high.

**Conclusions:**

In conclusion, the LEAD cohort has been established following high quality standards; it is representative of the Austrian population and offers a platform to understand lung development and ageing as a key mechanism of human health both in early and late adulthood.

## INTRODUCTION

Chronic respiratory diseases, such as chronic obstructive pulmonary disease (COPD) and asthma, are among the most prevalent, severe and costly human diseases. COPD currently is the 4^th^ cause of death worldwide and it is expected to be the 3^rd^ in a decade.^1^ It affects about 10% of the adult population in European countries. However, estimates in Austria almost triple that figure.^2^^,^^3^ Tobacco smoking is the main risk factor for COPD in developed countries but recent epidemiological studies indicate that about a quarter of individuals with chronic airflow limitation are never-smokers, and that early life events can also contribute to its pathogenesis.^4^ The influence of other environmental exposures either at work or at home, in urban and rural populations, is unclear.^5^ Likewise, asthma is the most prevalent chronic disease in childhood and affects about 5% of the population (children and adults).^6^ It is estimated that there are 300 million asthma patients worldwide.^7^

Recently, attention has focused on lung developmental issues during pregnancy, infancy, and adolescence as important drivers of respiratory diseases later in life.^8^ Both genetic^9^ and environmental factors, such as active^10^ and passive smoking,^11^ environmental pollution,^12^ recurrent bronchopulmonary infections,^13^ socioeconomic status,^14^ occupation, and diet, can alter normal lung. Low birth-weight is associated with lower lung function later in life.^15^^,^^16^ Yet, the potential elements and pathogenic mechanisms that control and influence lung development are not well established.^13^^,^^17^^,^^18^ The Framingham cohort study, among others, has shown that suboptimal lung function is the best predictor of respiratory and, of note, cardiovascular health during adulthood,^19^ but the relationship between respiratory health and other health conditions is unclear.^20^

Non-communicable diseases (NCDs) are the major global health problem of the XXI century.^21^ NCD’s are caused by complex gene-environment interactions across the lifespan, from fetus to old age^22^^,^^23^ and include, among others, chronic respiratory diseases, cardiovascular diseases, metabolic diseases, osteoporosis, and neuropsychiatric diseases. NCDs often coexist in the same patient.^24^ For instance, cardiovascular diseases, skeletal muscle dysfunction, osteoporosis, metabolic syndrome, and depression are highly prevalent in patients with COPD^25^ and contribute significantly to limit their quality of life and prognosis.^26^^–^^30^ Asthma is also associated with increased prevalence of comorbidities, albeit they frequently remain undiagnosed.^31^

The LEAD (Lung, hEart, sociAl, boDy) study (ClinicalTrials.gov; NCT01727518; http://clinicaltrials.gov) is a longitudinal, observational, population-based cohort that aims to investigate the relationship between genetic, environmental, developmental, and ageing factors influencing respiratory health and comorbidities through life. The general working hypothesis of LEAD is that the interaction of gene-environmental and socioeconomic factors influences lung development, the risk of occurrence of several major respiratory and cardiovascular diseases and other NCD’s, as well as their phenotypic (ie, clinical) presentation. Accordingly, the general goal of LEAD is to provide valid scientific information that contributes to better understand how genetic, environmental and socioeconomic factors influence: *(1)* the normal and pathologic lung growth, development and ageing (ie, the natural history of lung function in normal and pathological conditions); *(2)* the risk of development of major NCD’s like COPD, asthma, cardiovascular diseases, metabolic diseases (as diabetes and metabolic syndrome), osteoporosis, and neuropsychiatric diseases (mental disorders including anxiety, depression and impaired cognitive function); and, *(3)* the phenotypic heterogeneity and complex of chronic respiratory diseases, COPD in particular, and their relation with coexisting comorbidities. Within this general framework, different specific projects of LEAD will develop their own working hypothesis and specific goals in four domains (Lung, hEart, sociAl, boDy). Here, we describe in detail the methodology used and the external validity (ie, representativeness of the general Austrian population) of LEAD.

## METHODS

### Ethics

The local Ethics committee of Vienna approved the study (protocol number: EK-11-117-0711). Participants signed informed consent; those for children under the age of 18 had to be signed by their parents/legal representative.

### Study design

**Figure 1.**
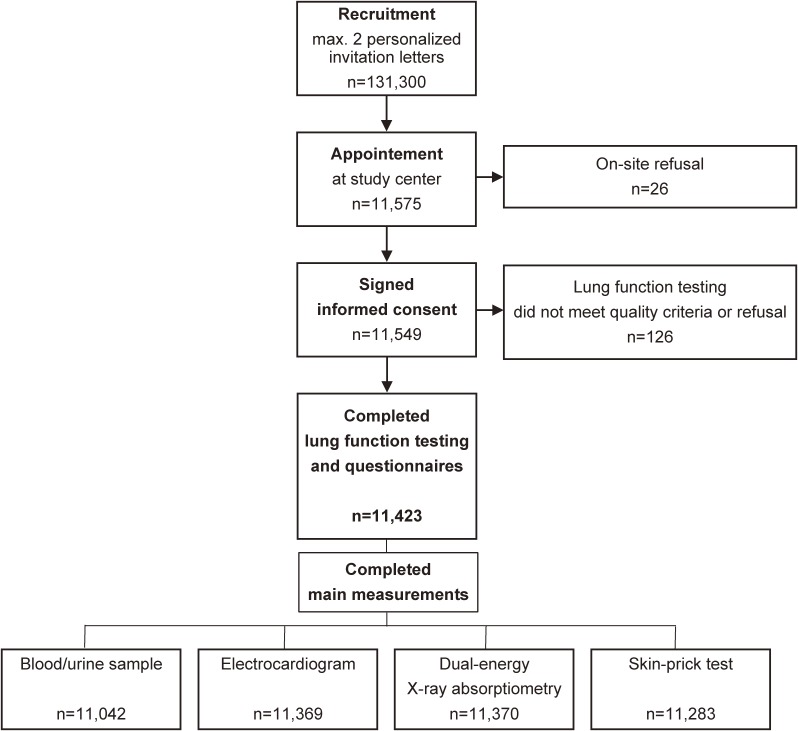
Flow chart of study population including invitation, inclusion, and main measurements.

LEAD (ClinicalTrials.gov; NCT01727518; http://clinicaltrials.gov) is a longitudinal, observational, population based cohort study with stratified samples from Vienna (urban population) and Lower Austria (rural population). Recruitment and first study visit started in February 2012 and finished in September 2016. A flow chart with numbers for initial recruitment, inclusion, and main measurements is presented as Figure 1. In total, 11,423 male and female, aged 6–80 years participated and completed the first visit, including main measurements as described in detail in Table 1.

**Table 1.** Measurements and methods used in LEAD

Measurement	Description	Equipment
**Demographics and Anthropometrics**		

	Age, Gender, Height, Weight, waist circumference	Questionnaire
	Socioeconomic status (Income, education, occupation, religion, migration)	Questionnaire
	Community (Urban/Rural)	
	height, weight, waist circumference	
Pregnancy test (female, 12–60 years)	Human chorionic gonadotropin (hCG) in urine	Alere HCG R-20™

**Birth and infancy data**		

	Birth-weight	Questionnaire
	Prematurity	Questionnaire
	Diseases and co-morbidities in family (non-communicable diseases, Allergy/Atopy)	Questionnaire
	Other: father/mother’s socioeconomic status, migration status, life-style, smoking history (pre-, peri- and post-pregnancy)	Questionnaire

**Exposures**		

	Individual smoking (Pack years, age start/quit smoking)	Questionnaire
	Environmental tobacco smoke (ETS): parents, grandparents, friends	
	Environmental tobacco smoke (ETS): at home, school, work	
	Data from the Environment Agency Austria of 17 monitoring units have been used in a calculated emission/combustion model for average concentrations of particulate matter 10 (PM10) and Nitrogen oxide (NO_X_) within 10 m^3^ of every participant’s home and work place address	Calculation model of the Institute for Internal Combustion Engines and Thermodynamics, Graz University of Technology, Austria and the Municipal Department for Environmental Protection of Vienna^48^

**Allergies**		

Skin-prick test^49^	Skin prick with allergens for ash tree, tree pollens mix (hazel, alder, birch), 5-grass-mix, mugwort, ragweed, ribwort, mites mix, Alternaria, dog and cat dander	ALK-Abelló Allergie-Service G.m.b.H.

**Domain Lung**		

Respiratory symptoms	· Wheezing	
	· Breathlessness	
	· Sputum	
	· Cough	
	Ever diagnoses of Asthma, COPD, other respiratory diseases	Questionnaire
Spirometry	Pre- and post-bronchodilation	Carl Rainer GmbH, Austria™
Body plethysmography	Pre- and post-bronchodilation	Carl Rainer GmbH, Austria™
Oscillatory resistance from visit 2	Post bronchodilation	Resmon™ Pro Forced Oscillation Technique
Diffusing capacity, DLCO from visit 2	Single-breath post bronchodilation	Carl Rainer GmbH, Austria™

**Domain hEart**		

	Ever diagnoses of angina pectoris, stroke, congestive heart failure, arrhythmia, other cardiovascular diseases	Questionnaire
Arterial blood pressure^50^	By Sphygmomanometer	Hokanson S12™ and DS400 Aneroid™
12-lead electrocardiogram^51^	Cardiac infarction injury score	Cardiosoft, GE Healthcare^®^, Austria
Non-invasive applanation tonometry^52^	Arterial stiffness by carotid femoral-pulse wave velocity (PWV) and Augmentation time Index by pulse wave analysis (PWA)	Spygmocor, Novomed^®^, Austria
Ankle-brachial index^53^	By sphygmomanometer and Doppler probe at upper and the lower extremities	ELCAT^®^ GmbH, Germany

**Domain SociAl**		

LEAD questionnaire	children (age 6–10 years)	Composed of: 1. National Health and Nutrition Examination Survey^54^ 2. ISAAC. The International Study of Asthma and Allergies in Childhood^55^ 3. American Thoracic European Community Respiratory Health Survey^56^
	adolescents (age 11–18 years)	
	adults (age 19–80 years)	

Anxiety and Depression	age 10–15 years	Children Anxiety and Depression scale^57^
	age >15 years	Hospital Anxiety and Depression Scale^58^
Quality of Life	by questionnaire	Study 12-item short form (SF-12) to assess health related quality of life^55^ Study 12-item short form (SF-12)^59^
Cognitive function	by questionnaire	
	age 15–70 years	Perceptual speed intelligence test
	age >70 years	Mini Mental Status Test (MMST)

**Domain boDy**		

Hand Grip Test^60^	Hand and forearm muscular strength	Trailite, TL-LSC100™
Dual-energy X-ray absorptiometry DXA®	Fat and lean mass (upper limp, trunk, android, gynoid, lower limp), Bone Mineral Density (lumbar spine, femur)^61^	Lunar Prodigy, GE Healthcare^®^, USA
	Activity, diet, nutrition^54^	Questionnaire
	Ever diagnoses of diabetes, osteoporosis, other metabolic diseases	Questionnaire

**Biobank**		

Fasting venous blood sample	Standard blood chemistry incl. differential, platelet count and Immunoglobulin E.	Samplosophy^®^ data matrix sample tube rack system ISO 9001:2008 certified quality management system
	Biobank storage at Biobank Medical University of Vienna 48 µl Frozen Serum, 40 µl Plasma, 20 µl Urine	

LEAD is directed by a Steering Committee, consisting of four Austrian academic clinical physicians (Marie-Kathrin Breyer, Robab Breyer-Kohansal, Sylvia Hartl, and Otto Burghuber). An international advisory board of five international respiratory experts (Alvar Agusti, Torben Sigsgaard, Michael Studnicka, Claus Vogelmeier, and Emiel Wouters) was convened. All of them discussed and agreed on the design of the study and interpretation of the results and have full access to the LEAD database.

### Recruitment strategy

For the urban cohort, we used the national inhabitants’ register to invite a randomized stratified sample (by age, gender, and residential area) of Vienna inhabitants to participate. For the rural cohort, due to the low total inhabitants’ number, every registered inhabitant of six villages from Lower Austria was invited. Selected urban and rural participants received a personalized invitation letter. If the selected person did not respond within 30 days, a maximum of two other personalized invitation letter were sent at regular intervals. Due to the national Austrian data protection act, we did not have access to information on other contact details (eg, telephone number). Exclusion criteria were pregnancy, current breast feeding, or poor German language skills. Individuals participated voluntarily without any reimbursement.

### Follow-up

Every participant will be assessed every 4 years from individual’s first study visit with all measurements described in detail in Table 1. In case of loss to follow-up, the national inhabitants’ register provides information on emigration or death. As the study is designed longitudinally, the numbers of loss to follow-up will be substituted with re-recruitment (by age, gender, and residential area).

### Measurements

All measurements were performed at the LEAD study centre of the Ludwig Boltzmann Institute for COPD and Respiratory Epidemiology at the Otto Wagner Hospital in Vienna, Austria. Table 1 details all measurements obtained in each participant according to her/his age. To screen for major NCD’s, a number of investigations and measurements were obtained in each of the four domains of the study (Lung, hEart, sociAl, boDy), as detailed in Table 1 and only briefly discussed below. Blood and urine samples were analyzed for routine clinical measurements and stored in a biobank for future investigations.

### Lung domain

Lung function measures included pre- and post-bronchodilation spirometry and static lung volumes, effort-independent measures of oscillatory resistance, and carbon monoxide lung diffusing capacity (DLCO). Measurements were obtained according to international recommendations^32^ and reference values used correspond to those of the Global Lung Function Initiative (GLI).^33^ Skin Prick Tests for major allergens (see Table 1) were obtained in every participant. Smoking history and exposure to environmental tobacco smoke (ETS) was recorded. History of respiratory diseases, allergy, and related medication from the individual and spouses, as well as respiratory symptoms, were collected using a questionnaire.

**Figure 2.**
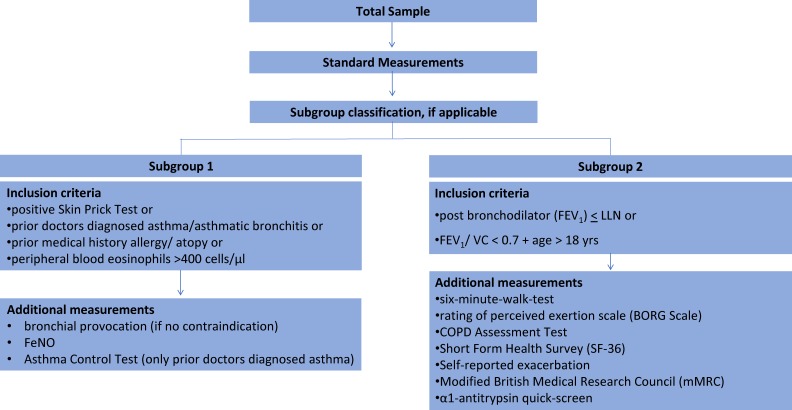
Flow chart for additional measurements. FeNO Fractional exhaled Nitric Oxide; FEV_1_ forced expiratory volume in the first second; VC Vital capacity; LLN lower limit of normal. References: Six-minute walking test (6 MWT)^62^ expressed as a percentage of the predicted distance^63^ and perceived dyspnoea using the modified BORG dyspnoea scale.^64^ CAT test; http://www.catestonline.org; 36-item short form (SF-36).^65^ ACT; https://www.asthmacontroltest.com/.

To investigate COPD and asthma in more detail, a subgroup of participants was invited for additional measurements. Every participant with a positive Skin Prick Test or doctor-diagnosed asthma or allergy or elevated blood eosinophils was re-invited for bronchial provocation and fractional exhaled nitric oxide (FeNO) testing and an Asthma Control Test^TM^ (subgroup 1). Every participant with an forced expiratory volume in the first second (FEV_1_)/forced vital capacity (FVC) below Lower Limits of Normal by GLI^33^ or below 70% was re-invited for a 6-minute walking test, COPD and health related questionnaires, and Alpha 1-Antitrypsin testing (subgroup 2). Details are explained in Figure 2.

### HEart domain

Cardiovascular measurements included arterial blood pressure, automated electrocardiogram, carotid femoral-pulse wave velocity, and blood pressure measurements at both the upper and the lower extremities. History of cardiovascular diseases and events and related medication from the individual and spouses were collected using a questionnaire.

### SociAl domain

**Figure 3.**
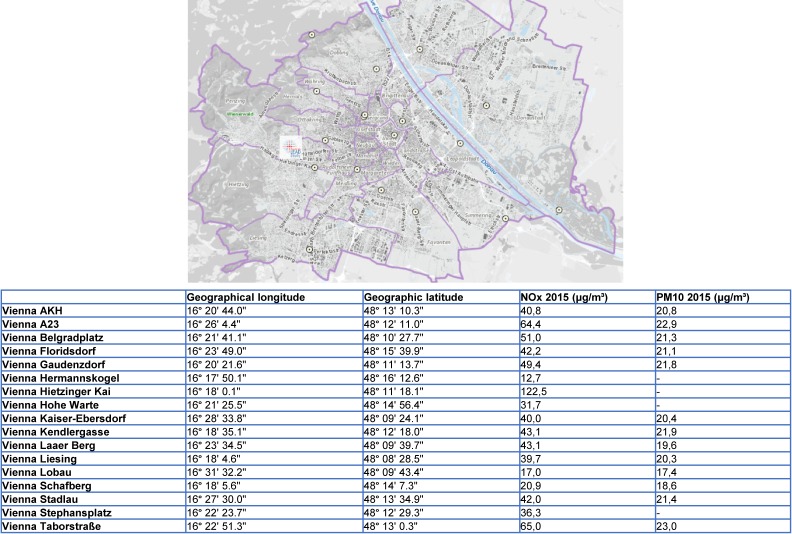
Monitoring units in Vienna, Austria and average PM 10 and NO_X_ in the year 2015 [Reference from the following source: City of Vienna, Municipal Department MA 22 - Environmental Protection].

To evaluate environmental risk factors for chronic respiratory diseases, data from the Environment Agency Austria of seventeen monitoring units was used to determine the average concentrations of particulate matter 10 (PM10) and Nitrogen oxide (NO_X_) within 10 m^3^ of every participant’s home and workplace. A map showing the monitoring units in Vienna and the average exposure on PM10 and NOx in the year 2015 is presented as Figure 3. Socioeconomic status (income, education, occupation) of the individual or parents/legal representative (if underage) was collected. To study the presence of neuropsychiatric diseases such as anxiety, depression, and impaired cognitive function, we used standardized questionnaires and test modules (as detailed in Table 1).

### BoDy domain

To determine the presence of diabetes and metabolic syndrome, we measured fasting glucose in peripheral venous blood, glycated hemoglobin (HbA1c), body mass index, waist circumference, fat mass/fat free mass, blood lipid profiles, and blood pressure. The presence of osteoporosis was determined using dual-energy X-ray absorptiometry. History of diabetes, metabolic syndrome, and osteoporosis and related medication from the individual and spouses were collected using a questionnaire.

### Quality control

To guarantee optimal quality of the whole process, the following measures were implemented from scratch: *(1)* all clinical examiners (medical students) were trained by senior staff (medical doctors/lung function technicians) and were supervised regularly; *(2)* standardized protocols were predetermined for every single measurement, based on international standards; *(3)* all questionnaires are interview based; *(4)* a web-tool was designed (www.linkthat.eu) that included control mechanism, which permits inconclusive data entry (eg, typing errors, implausible numbers), and all data from study equipment is uploaded directly by data transfer to avoid manual data input errors; *(5)* study monitoring is performed periodically and included extensive plausibility checks as well as regular monitoring reports of the interim data set; and, *(6)* special emphasis is placed on quality control of every measurement report, including a rigorous *post hoc* quality control of all original measurements by the steering committee.

### External validity

To determine the “representativeness” of the population included in LEAD compared to the Austrian population, we did extensive external validity testing. First, we compared LEAD results with those the most recent Austrian population data published by the Governmental Statistic Department in 2015.^34^ Second, the LEAD study population was compared with the Austrian Governmental Microcensus in terms of age, gender citizenship, educational level, and smoking status.^35^^,^^36^ The Microcensus is a randomly chosen Austrian household’s survey (quarterly sampled), in males and females aged 15–80 years, depicting socio-demographic details and, in a subsample, self-reported health status including smoking status. Due to the compulsory participation (denial punished by a fee) the Microcensus survey has a very high response rate (>99%) and high reliability.

### Statistical analysis

For this manuscript, continuous data are described using arithmetic means, standard deviations (SD) and ranges; binary and ordinal data by frequencies and percentages. In general, the level of significance (α) was set to 5% (Bonferroni-Holm correction applied whenever necessary). To analyze external validity (“representativeness”) α was set to 0.1% (99.9% confidence intervals) to prevent wrongly significant results for confidence intervals caused only because of the large sample sizes. All statistical analyses have been performed using SPSS 24.0 (IBM Corp, Armonk, NY, USA).^37^

In future analysis of specific LEAD projects we plan to use knowledge-driven and data-driven (unbiased) analysis, including principal component analysis, cluster analysis, and network analysis, to understand the complexity and heterogeneity of the cohort and different subgroup of participants.^22^^,^^38^^,^^39^

## RESULTS

### Participants’ characteristics

Table 2 presents the major baseline characteristics of the LEAD study cohort stratified by age. We included 1.344 children age 6 ≤ 18 years (male 54.4%) and 10,079 adults ≥18 years (male 46.65%). Smoking exposure was high since almost one fifth of children aged 6 ≤ 18 years had been exposed to environmental tobacco smoke and more than half of the adult participants were former or current smokers (56%). Former male smokers have a higher exposure to cigarette smoke compared to current smokers; therefore, we stratified all smokers using pack years (PY) showing that 19.5% have or have had a high cigarette consumption (>20 PY). All in all, most participants (86.9%) have any history of tobacco smoke exposure (passive and/or active).

**Table 2.** Baseline characteristics LEAD study cohort, stratified by age and gender

	Children (6–<18 years)		Adults (≥18 years)	
	*N* = 1,345		*N* = 10,078	
	
	Male	Female	Male	Female
	*N* = 732	*N* = 613	*N* = 4,701	*N* = 5,377
	
	Mean (SD) (*N* missing, if any)	Mean (SD) (*N* missing, if any)	Mean (SD) (*N* missing, if any)	Mean (SD) (*N* missing, if any)
Height, cm	152.3 (20.3)	149.6 (15.9)	177.5 (7.1)	164.2 (6.6)
Weight, kg	46.8 (19.3)	44.1 (15.3)	84.5 (14.2)	68.1 (14.0)
BMI, kg/m^2^	19.3 (4.1)	19.1 (3.9)	26.8 (4.3)	25.3 (5.2)
Waist circumference, cm	69.9 (12.4) (1)	68.3 (10.9) (1)	97.4 (12.7) (6)	88.5 (13.6) (14)

Smoking status	% [CI; 95%] (*N* missing, if any)	% [CI; 95%] (*N* missing, if any)	% [CI; 95%] (*N* missing, if any)	% [CI; 95%] (*N* missing, if any)

Never, %	96.6 [95.3–97.9]	97.1 [95.8–98.4]	38.1 [36.7–39.5]	49.1 [47.8–50.4]
Former, %	0.1 [0.0–0.3]	0.2 [0.0–0.6]	35.8 [34.4–37.2]	28.8 [27.6–30.0]
Pack years			22.8 (27.3) (10)	14.5 (18.7) (10)
Current, %	3.3 [2.0–4.6]	2.8 [1.5–4.1]	26.1 [24.8–27.4]	22.0 [20.9–23.1]
Pack years	1.3 (1.5) (2)	0.6 (0.9)	20.0 (21.3) (8)	16.0 (16.0) (10)
Former and currents smokers with <20 pack years, %	3.2 [1.9–4.5] (2)	2.9 [1.6–4.2]	36.8 [35.4–38.2] (18)	36.1 [34.8–37.4] (20)
Former and currents smokers with ≥20 pack years, %	none	none	25.0 [23.8–26.2] (18)	14.6 [13.7–15.5] (20)
Environmental tobacco smoke (ETS) at most days and/or nights of week for several hours				
In adolescents, %	17.8 [15.0–20.6] (2)	19.7 [16.6–22.8]	38.2 [36.8–39.6]	39.4 [38.1–40.7]
In adulthood, %	—	—	46.4 [45.0–47.8] (10)	49.5 [48.2–50.8] (7)

BMI, body mass index; CI, confidence interval; SD, standard deviation.

### External validity

**Figure 4.**
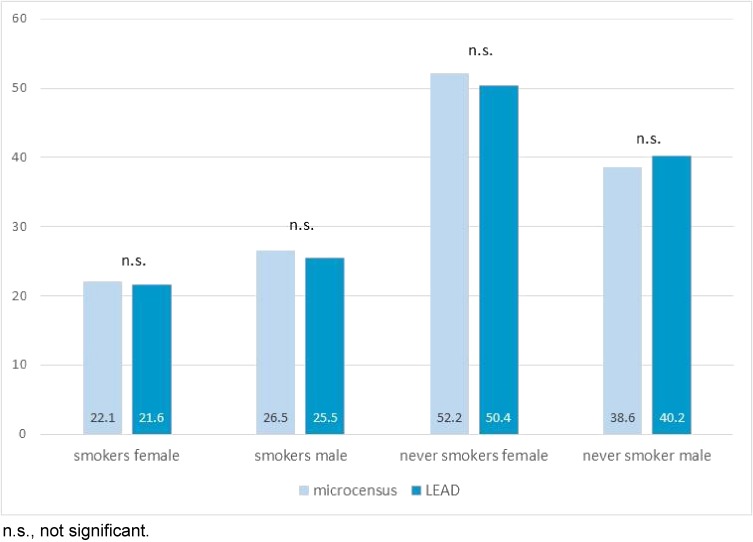
Smoking status LEAD study cohort compared to microcensus.

Compared to other epidemiological studies,^40^ the overall response rate in LEAD was low (total 8.7%, male: 7.7%, female 9.8%). The low participation rate is probably related to the very rigorous Austrian data protection law that prohibits iterative invitations and/or telephone contact. The LEAD study is a single-centered investigation, with participants having long travel to have examinations (no monetary incentive). As residential area was part of the recruitment strategy, a homogenous weighted proportional population sample of various Viennese districts had to be guaranteed. When comparing participation rates between Lower Austria, districts far away and nearby the LEAD study center, no difference were found (8.7% vs 6.5% vs 7.1%; all *P*-values non-significant). It is known, that participation rates in health surveys decreased over the past decades,^40^ and the considered personal benefit participating in a health survey in Austria may be low due to the high provision of health care. Despite this, the external validity (“representativeness”) of the LEAD cohort is very high. Table 3 shows that the demographic characteristics of LEAD participants are almost identical to those of the general Austrian population, stratified by age and gender. On the other hand, Table 4 compares the sociodemographic data (citizenship and educational level) of the LEAD cohort (15–80 years) with that of the Austrian Governmental Microcensus (15–80 years). The LEAD cohort was very similar in terms of citizenship but not in terms of educational level, which was shifted towards higher education. Finally, it is important to note that the smoking history of the LEAD study cohort was well matched with the results from the Austrian Microcensus both, in male and female participants (Figure 4).

**Table 3.** LEAD study cohort compared to Austrian population, stratified by age and gender

Age groups (years)	MALE				FEMALE			
	
	LEAD cohort *n* = 5,433		Austrian population *n* = 3,883,829	*P*	LEAD cohort *n* = 5,990		Austrian population *n* = 3,932,930	*P*
			
	*n*	% [99.9% CI]	%		*n*	% [99.9% CI]	%	
6–<10	306	5.6 [4.3–6.9]	4.3	n.s.	233	3.9 [3.0–4.8]	4.1	n.s.
10–<20	546	10.0 [8.6–11.4]	11.8	≤0.01	522	8.7 [7.4–10.0]	10.8	≤0.01
20–<30	698	12.8 [11.2–14.4]	15.1	≤0.01	806	13.5 [12.0–15.0]	14.2	n.s.
30–<40	756	13.9 [12.3–15.5]	15.0	n.s.	784	13.1 [11.6–14.6]	14.5	n.s.
40–<50	885	16.3 [14.5–18.1]	16.7	n.s.	1.077	18.0 [16.3–19.7]	16.5	n.s.
50–<60	877	16.1 [14.4–17.8]	16.8	n.s.	1.034	17.3 [15.6–19.0]	16.7	n.s.
60–<70	810	14.9 [13.2–16.6]	11.5	≤0.01	967	16.1 [14.4–17.8]	12.4	≤0.01
≥70	555	10.2 [8.8–11.6]	8.9	n.s.	567	9.5 [8.1–10.9]	10.9	n.s.

CI, confidence interval; n.s., not significant; *P*, *P*-value.

**Table 4.** Sociodemographics of LEAD study cohort compared to Microcensus

	LEAD cohort		Austrian Microcensus	
	
	*n*	% [99.9% CI]	% [99.9% CI]	*P*
Citizenship				
Austria	10,099	88.5 [87.5–89.5]	88.6 [87.4–89.8]	n.s.
Foreign	1,307	11.5 [10.5–12.5]	11.4 [10.2–12.6]	n.s.
Missing	17			
Education	LEAD cohort 15–80 years (*n* = 10.420)			
No education	182	1.7 [1.5–2.0]	1.1 [0.7–1.5]	≤0.01
Primary/lower secondary education	1,317	12.7 [12.0–13.3]	22.9 [21.3–24.5]	≤0.01
Finished apprenticeship/ vocational training	3,269	31.4 [30.5–32.3]	50.6 [48.7–52.5]	≤0.01
High school	2,769	26.6 [25.8–27.5]	14.0 [12.7–15.3]	≤0.01
University level	2,867	27.6 [26.7–28.4]	11.4 [10.2–12.6]	≤0.01
Missing	16			

CI, confidence interval; n.s., not significant; *P*, *P*-value.

### Limitations

1. To obtain all measurements in the entire study population including valid lung function testing (bodyplethysmography in particular) for proper comparability throughout age groups, only participants ≥6 years could be included. In addition, radiation exposure by dual-energy X-ray absorptiometry is prohibited in those <6 years of age. Therefore, first infant years of lung function development will not be available in this study.

2. Information on individual’s early life exposure like birth history, childhood respiratory infections, diagnoses, and maternal/paternal data on occupational exposure, migration, life style, and smoking behavior are documented only via questionnaires and retrospectively in those <18 years. The validity is demonstrated by the accompanying parents at the study visit. In those >18 years this information may be lacking due to individual’s memory (gap) or nescience.

3. The LEAD cohort shows higher education levels compared to the general Austrian population. This is a well-known challenge within populational based studies based on voluntary participation.^41^ Education is a well described and recognized factor on lung function, in both childhood and adulthood.^42^ This weakness is addressed as a major limitation within the LEAD cohort. To at least reduce this effect the authors will define recommended individual’s (for adults) and mothers/fathers (for children) socioeconomic status, including education, occupation, and income.^43^

## DISCUSSION

The ongoing LEAD study includes a large and carefully characterized cohort representative for the general population of Austria with respect to age, gender, and smoking status. The high quality of the dataset is guaranteed by the protocol based standard measurements, the trained personnel and interview based questionnaires obtained in all participants, through the direct data transfer from the equipment into the study specific tailored web tool preventing manual data input errors, and through regular study monitoring reports, interim data sets, and plausibility checks. In addition, rigorous *post hoc* quality control of every single measurement report is done by the steering committee.

The representativeness of the LEAD study cohort is supported by the comparison with the governmental data of the Austrian population that shows almost identical distribution of age, gender and citizenship. The shift towards higher education levels in the LEAD study cohort is also seen in other epidemiological studies, and is likely due to an increased health awareness of higher educated participants.^44^

The LEAD study has some clear strengths: *(1)* to our knowledge, it is among the biggest longitudinal population-based cohort studies providing pre- and post-bronchodilation spirometry and body plethysmography and diffusing capacity results to better characterize chronic respiratory disease as recommended by the European Respiratory Society^45^; *(2)* to validate prospectively lung function trajectories from infancy to late adulthood,^4^ the LEAD cohort included participants from 6 to 80 years, and most of the risk factors recently discussed^13^^,^^46^^,^^47^ have been considered in the analysis; *(3)* to study the impact of environmental factors on lung function, data from seventeen environmental monitoring units in Vienna calculate the average concentrations of particulate matter 10 (PM10) and Nitrogen oxide (NO_X_) within 10 m^3^ of every participant’s home and work place address using the emission and combustion model^48^; *(4)* to cover risk factors based on symptoms, socioeconomic status, smoking status, and self-rated general health, a LEAD study cohort tailored questionnaire was generated for different age groups based on validated questionnaires (for detailed information see Table 2). In addition, anxiety and depression, quality of life, and cognitive function were also evaluated; *(5)* to minimize recall bias for undiagnosed or self-reported coexisting diseases, the LEAD study measures key organ function data to screen for cardiovascular, metabolic, and body composition data for muscle/fat distribution as well as for osteoporosis and screening test for cognitive function. This approach provides a framework for the exploration of the relationships between lung health, age, and multi-morbidity in a broader context; and, finally, *(6)* LEAD has created a blood/urine biobank, hosted by the Medical University of Vienna, which is open to future international collaborations to better understand genetic risk factors and biomarkers influencing lung function development and decline.

In summary, the LEAD cohort has been established following high quality standards; it is representative of the Austrian population and offers a platform to understand lung development and ageing as a key mechanism of human health both, in early and late adulthood.

## References

[1] Lopez AD, Shibuya K, Rao C, Chronic obstructive pulmonary disease: current burden and future projections. Eur Respir J. 2006;27(2):397–412. 10.1183/09031936.06.00025805 16452599

[2] Douglass AB, Bornstein R, Nino-Murcia G, The Sleep Disorders Questionnaire. I: Creation and multivariate structure of SDQ. Sleep. 1994;17(2):160–167. 10.1093/sleep/17.2.160 8036370

[3] Schirnhofer L, Lamprecht B, Vollmer WM, COPD prevalence in Salzburg, Austria: results from the Burden of Obstructive Lung Disease (BOLD) Study. Chest. 2007;131(1):29–36. 10.1378/chest.06-0365 17218553

[4] Lange P, Celli B, Agusti A, Lung-function trajectories leading to chronic obstructive pulmonary disease. N Engl J Med. 2015;373(2):111–122. 10.1056/NEJMoa1411532 26154786

[5] Salvi S, Barnes PJ Is exposure to biomass smoke the biggest risk factor for COPD globally? Chest. 2010;138(1):3–6. 10.1378/chest.10-0645 20605806

[6] Pearce N, Sunyer J, Cheng S, Comparison of asthma prevalence in the ISAAC and the ECRHS. ISAAC Steering Committee and the European Community Respiratory Health Survey. International Study of Asthma and Allergies in Childhood. Eur Respir J. 2000;16(3):420–426. 10.1183/9031936.00.16337700 11028654

[7] Bousquet J, Kiley J, Bateman ED, Prioritised research agenda for prevention and control of chronic respiratory diseases. Eur Respir J. 2010;36(5):995–1001. 10.1183/09031936.00012610 20223919

[8] Boezen HM, Vonk JM, van Aalderen WM, Perinatal predictors of respiratory symptoms and lung function at a young adult age. Eur Respir J. 2002;20(2):383–390. 10.1183/09031936.02.00234102 12212971

[9] Jobe A. Fetal & Neonatal Lung Development; 2016.

[10] Kohansal R, Martinez-Camblor P, Agusti A, Buist AS, Mannino DM, Soriano JB The natural history of chronic airflow obstruction revisited: an analysis of the Framingham offspring cohort. Am J Respir Crit Care Med. 2009;180(1):3–10. 10.1164/rccm.200901-0047OC 19342411

[11] Lovasi GS, Diez Roux AV, Hoffman EA, Kawut SM, Jacobs DR Jr, Barr RG Association of environmental tobacco smoke exposure in childhood with early emphysema in adulthood among nonsmokers: the MESA-lung study. Am J Epidemiol. 2010;171(1):54–62. 10.1093/aje/kwp358 19942575 PMC2800303

[12] Gehring U, Beelen R, Eeftens M, Particulate matter composition and respiratory health: the PIAMA Birth Cohort study. Epidemiology. 2015;26(3):300–309. 10.1097/EDE.0000000000000264 25688676

[13] Svanes C, Sunyer J, Plana E, Early life origins of chronic obstructive pulmonary disease. Thorax. 2010;65(1):14–20. 10.1136/thx.2008.112136 19729360

[14] Jackson B, Kubzansky LD, Cohen S, Weiss S, Wright RJ A matter of life and breath: childhood socioeconomic status is related to young adult pulmonary function in the CARDIA study. Int J Epidemiol. 2004;33(2):271–278. 10.1093/ije/dyh003 15082626

[15] Edwards CA, Osman LM, Godden DJ, Campbell DM, Douglas JG Relationship between birth weight and adult lung function: controlling for maternal factors. Thorax. 2003;58(12):1061–1065. 10.1136/thorax.58.12.1061 14645976 PMC1746540

[16] Jones M Effect of preterm birth on airway function and lung growth. Paediatr Respir Rev. 2009;10(Suppl 1):9–11. 10.1016/S1526-0542(09)70005-3 19651391

[17] Tager IB, Hanrahan JP, Tosteson TD, Lung function, pre- and post-natal smoke exposure, and wheezing in the first year of life. Am Rev Respir Dis. 1993;147(4):811–817. 10.1164/ajrccm/147.4.811 8466114

[18] Hedlund U, Eriksson K, Ronmark E Socio-economic status is related to incidence of asthma and respiratory symptoms in adults. Eur Respir J. 2006;28(2):303–310. 10.1183/09031936.06.00108105 16540503

[19] Sorlie PD, Kannel WB, O’Connor G Mortality associated with respiratory function and symptoms in advanced age. The Framingham Study. Am Rev Respir Dis. 1989;140(2):379–384. 10.1164/ajrccm/140.2.379 2764375

[20] Sin DD, Man SF Why are patients with chronic obstructive pulmonary disease at increased risk of cardiovascular diseases? The potential role of systemic inflammation in chronic obstructive pulmonary disease. Circulation. 2003;107(11):1514–1519. 10.1161/01.CIR.0000056767.69054.B3 12654609

[21] Beaglehole R, Bonita R, Horton R, Priority actions for the non-communicable disease crisis. Lancet. 2011;377(9775):1438–1447. 10.1016/S0140-6736(11)60393-0 21474174

[22] Barabási AL, Gulbahce N, Loscalzo J Network medicine: a network-based approach to human disease. Nat Rev Genet. 2011;12(1):56–68. 10.1038/nrg2918 21164525 PMC3140052

[23] Christensen K, Doblhammer G, Rau R, Vaupel JW Ageing populations: the challenges ahead. Lancet. 2009;374(9696):1196–1208. 10.1016/S0140-6736(09)61460-4 19801098 PMC2810516

[24] WHO. 2008–2013 Action Plan for the Global Strategy for the Prevention and Control of Noncommunicable Diseases.

[25] Rabe KF, Hurd S, Anzueto A, Global strategy for the diagnosis, management, and prevention of chronic obstructive pulmonary disease: GOLD executive summary. Am J Respir Crit Care Med. 2007;176(6):532–555. 10.1164/rccm.200703-456SO 17507545

[26] Wouters EF COPD: from an organ- to a disease-oriented approach. COPD. 2008;5(2):73–74. 10.1080/15412550801940200 18415805

[27] Agusti A, Soriano JB COPD as a systemic disease. COPD. 2008;5(2):133–138. 10.1080/15412550801941349 18415812

[28] Eickhoff P, Valipour A, Kiss D, Determinants of systemic vascular function in patients with stable chronic obstructive pulmonary disease. Am J Respir Crit Care Med. 2008;178(12):1211–1218. 10.1164/rccm.200709-1412OC 18836149

[29] Breyer MK, Spruit MA, Celis AP, Rutten EP, Janssen PP, Wouters EF Highly elevated C-reactive protein levels in obese patients with COPD: a fat chance? Clin Nutr. 2009;28(6):642–647. 10.1016/j.clnu.2009.05.005 19540024

[30] Decramer M, De Benedetto F, Del Ponte A, Marinari S Systemic effects of COPD. Respir Med 2005;99(Suppl B):S3–S10. 10.1016/j.rmed.2005.09.010 16219454

[31] Su X, Ren Y, Li M, Zhao X, Kong L, Kang J Prevalence of comorbidities in asthma and nonasthma patients: a meta-analysis. Medicine (Baltimore). 2016;95(22):e3459. 10.1097/MD.0000000000003459 27258489 PMC4900697

[32] Miller MR, Hankinson J, Brusasco V, Standardisation of spirometry. Eur Respir J. 2005;26(2):319–338. 10.1183/09031936.05.00034805 16055882

[33] Quanjer PH, Stanojevic S, Cole TJ, ; Initiative ERSGLF Multi-ethnic reference values for spirometry for the 3–95-yr age range: the global lung function 2012 equations. Eur Respir J. 2012;40(6):1324–1343. 10.1183/09031936.00080312 22743675 PMC3786581

[34] Statisik Austria. https://www.statistik.at/web_de/statistiken/menschen_und_gesellschaft/bevoelkerung/bevoelkerungsstruktur/bevoelkerung_nach_alter_geschlecht/index.html. Assessed 12.4.2017.

[35] Statistik Austria. http://www.statistik.at/web_de/frageboegen/private_haushalte/mikrozensus/index.html. Assessed 12.4.2017.

[36] Statistik Austria. http://www.statistik.at/web_de/statistiken/menschen_und_gesellschaft/gesundheit/gesundheitsdeterminanten/rauchen/index.html. Assessed 12.4.2017.

[37] IBM Corp. Released 2016. IBM SPSS Statistics for Windows VA, NY: IBM Corp.

[38] Burgel PR, Paillasseur JL, Caillaud D, ; Initiative BSC Clinical COPD phenotypes: a novel approach using principal component and cluster analyses. Eur Respir J. 2010;36(3):531–539. 10.1183/09031936.00175109 20075045

[39] Diez D, Agusti A, Wheelock CE Network analysis in the investigation of chronic respiratory diseases. From basics to application. Am J Respir Crit Care Med. 2014;190(9):981–988. 10.1164/rccm.201403-0421PP 25254605

[40] Mindell JS, Giampaoli S, Goesswald A, ; Group HESRR Sample selection, recruitment and participation rates in health examination surveys in Europe—experience from seven national surveys. BMC Med Res Methodol. 2015;15:78. 10.1186/s12874-015-0072-4 26438235 PMC4595185

[41] Strandhagen E, Berg C, Lissner L, Selection bias in a population survey with registry linkage: potential effect on socioeconomic gradient in cardiovascular risk. Eur J Epidemiol. 2010;25(3):163–172. 10.1007/s10654-010-9427-7 20127393

[42] Antó JM, Vermeire P, Vestbo J, Sunyer J Epidemiology of chronic obstructive pulmonary disease. Eur Respir J. 2001;17(5):982–994. 10.1183/09031936.01.17509820 11488336

[43] Diemer MA, Mistry RS, Wadsworth ME, López I, Reimers F Best practices in conceptualizing and measuring social class in psychological research. Anal Soc Issues Public Policy. 2013;13(1):77–113. 10.1111/asap.12001

[44] Kohansal R, Soriano JB, Agusti A Investigating the natural history of lung function: facts, pitfalls, and opportunities. Chest. 2009;135(5):1330–1341. 10.1378/chest.08-1750 19420200

[45] Bakke PS, Ronmark E, Eagan T, ; European Respiratory Society Task Force Recommendations for epidemiological studies on COPD. Eur Respir J. 2011;38(6):1261–1277. 10.1183/09031936.00193809 22130763

[46] Grigg J Suppression of lung growth by environmental toxins. Thorax. 2016;71(2):99–100. 10.1136/thoraxjnl-2015-207892 26719228

[47] Martinez FD Early-life origins of chronic obstructive pulmonary disease. N Engl J Med. 2016;375(9):871–878. 10.1056/NEJMra1603287 27579637

[48] Kurz C, Orthofer R, Sturm P, Projection of the air quality in Vienna between 2005 and 2020 for NO2 and PM10. Urban Climate. 2014;10:703–719. 10.1016/j.uclim.2014.03.008

[49] American Academy of Allergy AaIACoA, Asthma and Immunology Allergy diagnostic testing: an updated practice parameter. Ann Allergy Asthma Immunol. 2008 Mar;100(3)(Suppl 3):S1–S148. 10.1016/S1081-1206(10)60305-5 18431959

[50] Pickering TG, Hall JE, Appel LJ, Recommendations for blood pressure measurement in humans and experimental animals: part 1: blood pressure measurement in humans: a statement for professionals from the Subcommittee of Professional and Public Education of the American Heart Association Council on High Blood Pressure Research. Circulation. 2005. 10.1161/01.CIR.0000154900.76284.F6 15699287

[51] Rautaharju PM, Warren JW, Jain U, Wolf HK, Nielsen CL Cardiac infarction injury score: an electrocardiographic coding scheme for ischemic heart disease. Circulation. 1981;64(2):249–256. 10.1161/01.CIR.64.2.249 7249294

[52] Laurent S, Cockcroft J, Van Bortel L, ; European Network for Non-invasive Investigation of Large Arteries Expert consensus document on arterial stiffness: methodological issues and clinical applications. Eur Heart J. 2006;27(21):2588–2605. 10.1093/eurheartj/ehl254 17000623

[53] European Stroke Organisation, Tendera M, Aboyans V, Bartelink ML, ; ESC Committee for Practice Guidelines ESC Guidelines on the diagnosis and treatment of peripheral artery diseases: document covering atherosclerotic disease of extracranial carotid and vertebral, mesenteric, renal, upper and lower extremity arteries: the Task Force on the Diagnosis and Treatment of Peripheral Artery Diseases of the European Society of Cardiology (ESC). Eur Heart J. 2011;32(22):2851–2906. 21873417 10.1093/eurheartj/ehr211

[54] National Health and Nutrition Examination Survey. https://wwwn.cdc.gov/nchs/nhanes/Default.aspx. Assessed 2010.

[55] ISAAC. International Study of Asthma and Allergies in Childhood. http://isaac.auckland.ac.nz/resources/tools.php?menu=tools1#quest. Assessed 2010. 10.1002/ppul.20525 17123321

[56] European Community Respiratory Health Survey. http://www.ecrhs.org/quests.htm. Assessed 2010.

[57] Stiensmeier-Pelster J, Braune-Krickau M, Schürmann M, Duda K. DIKJ. Depressions-Inventar für Kinder und Jugendliche. 2000.

[58] Zigmond A, Snaith RP The hospital anxiety and depression scale. Acta Psychiatr Scand. 1983;67(6):361–370. 10.1111/j.1600-0447.1983.tb09716.x 6880820

[59] Ware J, Kosinski M, Keller SD A 12-Item Short-Form Health Survey: construction of scales and preliminary tests of reliability and validity. Med Care. 1996;34:220–233. 10.1097/00005650-199603000-00003 8628042

[60] Fess EE. In: Casanova JS, ed. *Clinical assessment recommendations*. 2. Chicago: American Society of Hand Therapists; 1992. Grip strength; pp. 41–45.

[61] Looker AC, Wahner HW, Dunn WL, Updated data on proximal femur bone mineral levels of US adults. Osteoporos Int. 1998;8(5):468–489. 10.1007/s001980050093 9850356

[62] ATS Committee on Proficiency Standards for Clinical Pulmonary Function Laboratories ATS statement: guidelines for the six-minute walk test. Am J Respir Crit Care Med. 2002;166(1):111–117. 10.1164/ajrccm.166.1.at1102 12091180

[63] Troosters T, Gosselink R, Decramer M Six minute walking distance in healthy elderly subjects. Eur Respir J. 1999;14(2):270–274. 10.1034/j.1399-3003.1999.14b06.x 10515400

[64] Borg GA Psychophysical bases of perceived exertion. Med Sci Sports Exerc. 1982;14(5):377–381. 10.1249/00005768-198205000-00012 7154893

[65] Mahler DA, Mackowiak JI Evaluation of the short-form 36-item questionnaire to measure health-related quality of life in patients with COPD. Chest. 1995;107(6):1585–1589. 10.1378/chest.107.6.1585 7781351

